# Standing-Posture Recognition in Human–Robot Collaboration Based on Deep Learning and the Dempster–Shafer Evidence Theory

**DOI:** 10.3390/s20041158

**Published:** 2020-02-20

**Authors:** Guan Li, Zhifeng Liu, Ligang Cai, Jun Yan

**Affiliations:** 1 Institute of Advanced Manufacturing and Intelligent Technology, Beijing University of Technology, Beijing 100022, China; b201401001@emails.bjut.edu.cn; 2 Beijing Key Laboratory of Advanced Manufacturing Technology, Beijing University of Technology, Beijing 100022, China; 3 North China Institute of Science and Technology, Langfang 065201, China; 4 Mechanical Industry Key Laboratory of Heavy Machine Tool Digital Design and Testing, Beijing 100022, China

**Keywords:** standing-posture recognition, convolutional neural network, HRC, machine learning, data fusion

## Abstract

During human–robot collaborations (HRC), robot systems must accurately perceive the actions and intentions of humans. The present study proposes the classification of standing postures from standing-pressure images, by which a robot system can predict the intended actions of human workers in an HRC environment. To this end, it explores deep learning based on standing-posture recognition and a multi-recognition algorithm fusion method for HRC. To acquire the pressure-distribution data, ten experimental participants stood on a pressure-sensing floor embedded with thin-film pressure sensors. The pressure data of nine standing postures were obtained from each participant. The human standing postures were discriminated by seven classification algorithms. The results of the best three algorithms were fused using the Dempster–Shafer evidence theory to improve the accuracy and robustness. In a cross-validation test, the best method achieved an average accuracy of 99.96%. The convolutional neural network classifier and data-fusion algorithm can feasibly classify the standing postures of human workers.

## 1. Introduction

**Figure 1:**
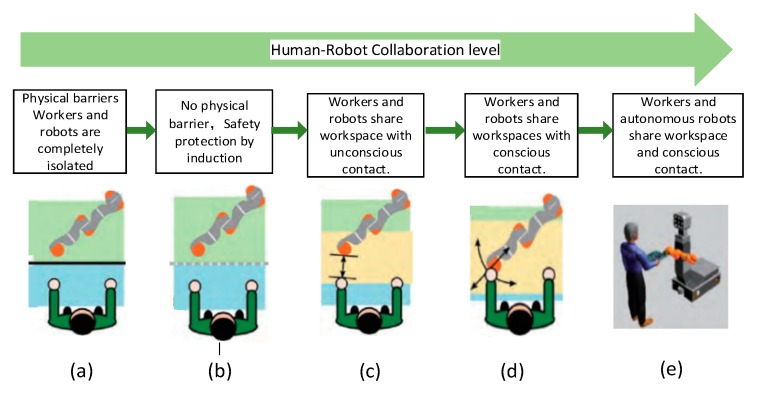
Development of human–robot cooperation.

With the rapid development of robots and artificial intelligence technology, manufacturing has increasingly relied on human–robot collaborations (HRCs). In future manufacturing industries, robots and humans will collaborate in processing a product at the same workstation [1,2]. To improve the flexibility, production efficiency, and quality of this collaboration, robots must perceive the environment in real time and accordingly adapt to environmental changes [3]. HRC has been developed through five main levels as described below (see also Figure 1):

(a) At the lowest level of collaboration, humans and machines are separated by a protective fence, and work in their own workspaces.

(b) At the next level of collaboration, no guardrails are installed but inductive safety protection is installed. 

(c) At the middle level, humans and robots share part of the workspace, and are unconsciously contacted. 

(d) At the second-highest level, humans and robots share the same workspace and are consciously contacted, but the robot is fixed.

(e) At the highest level of collaboration, people and robots share the same workspace, and the robots can move and perceive human actions in real time [4,5,6]. 

HRC will become a key technology in future intelligent manufacturing. In an HRC system, humans and robots will cooperate on the same tasks in the same workspace [7]. Therefore, the robots must recognize human actions and understand human intentions, which poses a very challenging problem.

The robot in an HRC system must make different decisions. For example, in an assembly scenario, the assembly parts should be correctly recognized and accurately installed at the target location. The reliability of the robot’s decisions is degraded by limitations in the detection systems and disturbances [8]. As human workers, robots, the environment, and other components of a particular situation often change rapidly, modeling and planning collaboration tasks for humans and robots in an unstructured environment is a challenging task. Michalos et al. [7] presented the implementation of a robotic system for advanced human–robot collaboration assembly and discussed all the technological approaches that have been implemented for facilitating the interaction and support of human operators. Human postures by robots can be broadly perceived as contacting postures (requiring sensors for touching the human body) and non-contacting postures (not requiring sensors that contact the human body). In the non-contacting category, human postures are mainly perceived by sensors such as Red–Green–Blue cameras [9], infrared, or laser sensors [10]. In one human–robot interaction and cooperation system, human postures were acquired by a depth camera [11,12,13]. Wang et al. proposed a novel methodology of real-time active collision avoidance in an augmented environment, in which monitoring and collision detection was performed by virtual three-dimensional models of robots and real camera images of operators [14]. Human actions can be recognized by inertial measurement unit sensors worn on the person [15,16]. Some scholars have used contact-force perception in human–robot interactions and collaborations. For example, a manipulator can be flexibly controlled by installing force sensors at the end of the manipulator [17]. Intelligent sensors that detect forces beneath the skin have achieved flexible and safe HRCs [18,19]. Pressure sensors that recognize various human motion postures—sitting, standing, and lying—have been processed into pressure arrays and embedded in cushions, carpets, and mattresses [20,21,22,23]. Human-based assembly operations have also been monitored through machine learning [24].

Currently, human footprints are detected and identified by a pressure-sensor matrix. Human-posture recognition in HRC requires further development. To this end, the present article attempts to recognize the standing postures of individual human workers in HRC scenes. The core contributions of this paper are as follows:

Standing-posture classification system (SPCS): We propose a low-cost surface-pressure-based SPCS (Section 3) that actively perceives human postures in HRC, and provides more effective data for human–computer interactions and human security.

Classification method on human standing posture: We divide human standing postures into nine categories and classify them by an optimized seven-layer convolutional neural network (CNN). To improve the recognition rate, we fuse the outputs of the CNN classifier, support vector machine (SVM) classifier, and *k*-nearest neighbor (KNN) classifier using the Dempster–Shafer (D–S) evidence theory (CSK–DS method).

## 2. Related Work

This section briefly overviews previous studies on human-posture recognition based on pressure-sensor matrices, then introduces CNN applications in pressure-image classification. 

Human-posture perception based on pressure matrices has been rarely applied in daily life scenarios and industrial environments. Human activities in daily life scenes have been detected by an intelligent textile-based sensing surface [25] that perceives not only the human body, but also various objects. Walking footsteps are detected by a low-cost intelligent carpet system. Seven gait features have been extracted from the piezo-resistance change profile formed by humans walking on the carpet [26]. A large-area pressure-sensitive floor recognizes footprints [26] and behaviors [27] through target segmentation, target tracking, and target recognition.

Recent experiments have transferred the force of a two-dimensional pressure-sensor mat into a pressure image. Pressure-image analysis by image-feature extraction and classification is a new trend in current research [28,29]. The coordinates and pressure values at the maximum and central pressure points in different areas of the plantar have been extracted as Laplace spectral features from barefoot static-plantar pressure images, and used in access control and attendance systems [30]. The biological characteristics of human footsteps have been identified by measuring instruments installed in a floor [9]. Video and floor pressure data have been fused into a multimodal gesture-recognition framework that improves the recognition of visually ambiguous gestures [20].

In the last few years, deep learning methods have been shown to outperform previous state-of-the-art machine learning techniques in several fields [31], including recognition of human actions. CNN is more effective in applications than many traditional classification methods [32]. Recognizing that sitting on a chair in an awkward posture or for long periods is a risk factor for musculoskeletal disorders, Kim [33] proposed a monitoring system that classifies children’s sitting postures by machine-learning algorithms. Costilla-Reyes et al. [34] proposed a model that learns spatial footstep features and recognizes footsteps by a nonlinear SVM classifier. Zhou et al. [35] presented a person-identification approach based on the morphing of footsteps measured by a fabric-based pressure-mapping sensor system. Features extracted by transfer learning have also been applied in person-identification tasks [28]. D–S evidence theory has been widely used in information fusion, uncertain reasoning, and other fields. It has a solid mathematical foundation and obtains good fusion results by a simple reasoning form without prior probability [36,37]. Despite these achievements, human-posture recognition remains insufficient for industrial HRC systems. In particular, the flexibility and stability of HRC systems require further study, and the safety of humans should be fully guaranteed [7]. Therefore, the study of human motion and intention perception is far from complete.

## 3. Methods 

### 3.1. Selected Standing Postures

**Figure 2:**
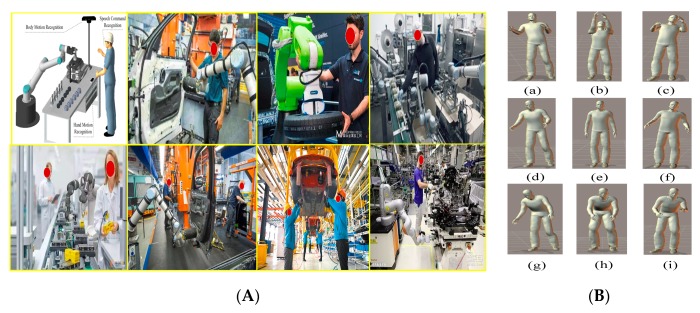
(**A**) Some actual scenes of human–robot cooperation. (**B**) Standing postures in the experiment: (**a**) right-backward standing (RBS); (**b**) back-incline standing (BIS); (**c**) left-backward standing (LBS); (**d**) right-leaning standing (RLS); (**e**) upright standing (URS); (**f**) left-leaning standing (LLS); (**g**) right-forward standing (RFS); (**h**) forward-leaning standing (FLS); (**i**) left-forward standing (LFS).

In an actual HRC environment, humans usually stand throughout the HRC process (see Figure 2A). Moreover, different standing postures correspond to different actions in the workflow. Therefore, we consider nine typical standing postures of the human body in an HRC scenario. The standing-action states are divided into nine classes, each corresponding to one posture (Figure 2): (a) right-backward standing (RBS); (b) back-incline standing (BIS); (c) left-backward standing (LBS); (d) right-leaning standing (RLS); (e) upright standing (URS); (f) left-leaning standing (LLS); (g) right-forward standing (RFS); (h) forward-leaning standing (FLS); and (i) left-forward standing (LFS).

### 3.2. Standing Postures Classification System

Our proposed SPCS consists of two parts: a pressure-sensing floor, and a data collecting unit.

**Figure 3:**
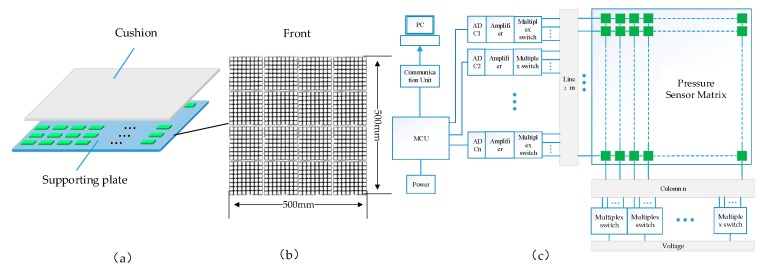
Standing-posture classification system (SPCS) framework: (**a**) structure of the pressure floor; (**b**) sensor matrix; (**c**) data acquisition system.

(1) Pressure-sensing floor. The pressure-sensing floor (Figure 3a) is composed of three layers: a pressure buffer layer, a pressure-sensor array, and a supporting plate. The buffer layer is a 3 mm-thick cushion with an elastic property, abrasion resistance, smooth surface, and the ability to regain its original state after the pressure is canceled. With these characteristics, the cushion can effectively and continuously transmit the pressure of the human body while protecting the pressure sensor, thereby ensuring a uniform surface of the pressure-sensing matrix. The middle layer (i.e., the pressure-sensor array) perceives the pressure transmitted by the buffer layer close to the upper surface of the supporting floor. Meanwhile, the surface finish of the bottom support ensures uniform force detection by the film pressure sensor. The pressure-sensor array has 32 rows and 32 columns distributed over a measuring area of (500 × 500) mm^2^, as shown in Figure 3b. The sensitivity range of a single-point sensor was selected as 0–25 kg, suitable for a 100-kg person standing on the pressure-sensing floor. The bottom plate is composed of rigid support material with a smooth upper surface and sufficient hardness to resist deformation under normal pressures of the human body.

(2) Data acquisition system. A human worker standing on the floor generates a foot-pressure distribution over the sensor matrix, which is converted to a greyscale image by the data acquisition system of the SPCS (Figure 3c). The data acquisition system is mainly divided into two parts: the field-data processing unit for signal acquisition and amplification, and the host computer software. The signal produced by the pressure floor is connected to an STM32 [38] family of a 32-bit microcontroller unit (MCU, STM32F103ZET6). The MCU has 16 analog-to-digital converters (ADC) channels with 12-bit precision. Through a high-speed analog switch chip, 16 × 64 pressure-sensing data can be collected by a cyclic scanning algorithm. The data acquisition frequency is 40 Hz.

### 3.3. Participants

The pressure data were collected from 10 experimental subjects (8 male and 2 female students attending Beijing University of Technology, Beijing, China). The subjects’ weights ranged from 41 to 96 kg, roughly covering the weight range of human workers in China. The detailed information of the participants is given in Table 1. We designed a data acquisition process and a set of predefined standing postures. At least 100 samples of each posture were collected from each subject. Prior to data acquisition, all subjects were trained to perform different actions under our instructions. During the data collection, each simple human activity was performed within 5 s. We obtained a group of static pressure images of the human body. After completing the posture data collection, the activity state was assessed by the program.

### 3.4. Classification Model of Standing Posture

#### 3.4.1. CNN Structure

**Figure 4:**
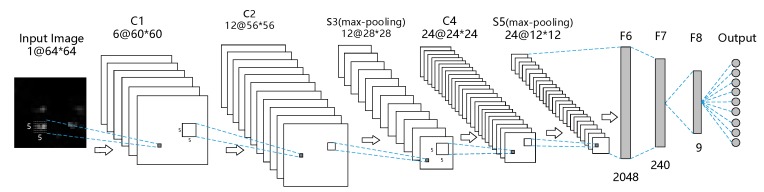
Structure of the convolutional neural network (CNN) in the posture-recognition model.

The resolution of pressure image is 32 × 32, while that of the handwritten dataset in MNIST is 28 × 28. Lenet-5 has achieved very high results in the field of handwriting recognition, therefore a network structure similar to Lenet-5 was used to classify the human standing pressure images. Figure 4 shows the basic structure of CNN that recognizes the pressure images of human standing postures. The network contains three convolutional layers (C1, C2, and C4), two pooling layers (S3 and S5), three fully connected layers (F6, F7, and F8), and a classification layer. The first six layers perform the feature extraction, and the final layer classifies the postures. Each convolutional layer is followed by a batch normalization (BN) layer, an activation function layer, and a dropout layer. To optimize the network performance, the batch size of each layer was set to 64. The activation function uses a rectified linear unit (ReLU): f(x)=max(0,x). Considering the real-time classification of the system, the output uses a Softmax regression classifier.

The cost function is defined as J(θ):

(1) $$J\left(\theta \right)=-\frac{1}{m}\left[\sum_{i=1}^{m}\sum_{j=1}^{k}I\left\{y^{\left(i\right)}=j\right\}\log\frac{e^{\theta_{j}^{T}x\left(i\right)}}{\sum_{j=1}^{k}e^{\theta_{j}^{T}x\left(i\right)}}\right]$$

The partial derivative of J(θ) for $\theta_{j}$:

(2) $$\frac{\nabla J\left(\theta \right)}{\nabla \theta_{j}}=\frac{1}{m}\left[\sum_{i=1}^{m}I\left\{y^{\left(i\right)}=j\right\}x^{\left(i\right)}-\frac{x^{\left(i\right)}e^{\theta_{j}^{T}x\left(i\right)}}{\sum_{j=1}^{k}e^{\theta_{j}^{T}x\left(i\right)}}\right]$$

In Equations (1) and (2), θ is model parameter, $\theta =[\theta_{1},\theta_{2},\cdots \theta_{k}]\in {\mathbb{R}}^{n+1}$, k represents the dataset with k classes, m represents the number of samples in each class, and θ represents the model parameters. $I\left\{y^{\left(i\right)}=j\right\}$ denotes that when $y^{\left(i\right)}$ belongs to the class j,$I\left\{y^{\left(i\right)}=j\right\}\;=\;1$, otherwise $I\left\{y^{\left(i\right)}=j\right\}\;=\;0$. The weights of the network are adjusted by the backpropagation (BP) algorithm. The whole network trains approximately 7146 K parameters.

After convolution and pooling, a 64 × 64-pixel image is converted to 30 feature maps, each of 64 × 64 pixels. After conversion to one-dimensional vectors, the feature maps are connected in the fully connected layer. The number of fully connected layer neurons is an important parameter in a network structure. From a feature-extraction viewpoint, the output of the fully connected layer is the high-level feature representation of the input image and is inserted as the input vector to the Softmax regression layer. After many comparative experiments, the number of neurons in the connective layer was decided to be 2048.

#### 3.4.2. Data Augmentation

Data augmentation [39] is a common technique for improving the original dataset. A deep learning application requires sufficiently many data to avoid the over-fitting problem. If the dataset is small, the positions in the image pixels can be changed by transforming the original image data without changing their features. Some suitable transformations are translation, rotation, and scaling. Rotation and translation simulate different orientations and floor locations of the human-body standing posture, respectively, and scaling the pixel values simulate different body weights. To prevent the effective pixels from moving out of the image boundary after a translation, we add eight zero-pixels to each edge of each image, obtaining 64 × 64-pixel images. The blank area after a translation is filled with pixels in the edge area. Finally, we obtained a dataset of 100 K pressure images.

### 3.5. Other Classifiers

To determine the best classification model in the proposed system, we applied the SVM, KNN, random forest (RF), decision tree (DT), Naive Bayes (NB), and BP neural network classifiers to the acquired dataset. SVM [40] is among the most popular and highest performing classifiers owing to its high generalization performance. In this study, a radial basis function (RBF) kernel function was chosen for the SVM. The RBF is the most widely used kernel function, delivering superior performance on both large and small datasets with fewer parameters than the polynomial kernel function. During training, the grid-search method was used to get the value of the super parameter: C = 0.3636, sigma = 0.7112. The KNN [41] is popularly used in data mining and statistics owing to its simple implementation and significant classification performance. In the KNN algorithm, the parameter *k* represents the number of neighbors. If *k* is small, it will cause overfitting, otherwise, the target cannot be classified. During the experiment, we choose 3, 5, 7, and 9 respectively. The test results show that when *k* = 5, the accuracy is the highest. NB [42] is a simple but practical classifier with a wide range of applications in face recognition, cancer diagnosis, and other fields. The DT algorithm is favored for its simplicity over other machine-learning classification algorithms. In the DT method, we adjusted the minimum parent size from 5 to 30 in 5-unit intervals. An RF [40] is a collection of decision trees learned on a random subset of training data. When the RF method was used to adjust parameters, the grid-search method was also used. We ascertained that the minimum number of trees delivering optimal performance is 30. Finally, in a data training process by the BP algorithm [41], we choose a foot’s pressure image (5 * 10) as the feature vector, so the input layer was 50, the output layer was 9, the number of hidden layers was selected from 25 to 70 and was selected for each interval of 5. We found that when the number of hidden layers was 55, the recognition rate was the highest.

### 3.6. D–S Evidence Theory and the Multi-Classifier Fusion Algorithm

#### 3.6.1. D–S Evidence Theory

This subsection introduces some basic concepts of the D–S evidence theory [36]. Let $\Theta =\left\{\theta_{1},\theta_{2},\cdots ,\theta_{n}\right\}$ be the set of all possible answers to the problem of human standing recognition. An object $\theta_{i}$ is a conclusion reached by the system. The important functions in D–S theory are the basic probability-assignment function, the belief function (BEL), and the likelihood function (PLS). These three functions are respectively defined as follows:

(3) $$\mathrm{BPA}:\sum_{A\in \Theta }m(A)=1,m(\phi )=0$$

(4) $$\mathrm{BEL}:2^{\Theta }\to [0,1],Bel(A)=\sum_{B\in A}m(B)$$

(5) $$\mathrm{PLS}:2^{\Theta }\to [0,1],Pl(A)=1-Bel(\bar{A})=\sum_{A\cap B\neq \phi }m(B)$$

In Equation (3), A is a hypothesis in the recognition framework A⊆Θ and m(A) is a basic probability-assignment function. In Equation (4), Bel(A) is the sum of the basic distribution probability functions of all subsets of A, and Pl(A) is the sum of the basic probability distributions of all subsets that intersect A. As the BELs are independent on the same recognition framework, they can be combined into a common agreement on a subset of $2^{\Theta }\to \left[0,1\right]$, and any conflicts can be quantified by Dempster’s combination rule. For all A⊆Θ and given n masses $m_{1},m_{2},\cdots m_{n}$, Dempster’s rule is calculated by Equations (6) and (7):

(6) $$m_{1}⊕m_{2}⊕\cdots ⊕m_{n}(A)=\frac{1}{K}\sum_{A_{1}\cap A_{2}\cap \cdots \cap A_{n}}m_{1}(A_{1})\cdot m_{2}(A_{2})\cdot \cdots \cdot m_{n}(A_{n})$$

(7) $$K=1-\sum_{A_{1}\cap A_{2}\cap \cdots \cap A_{n}=\phi }m_{1}(A_{1})\cdot m_{2}(A_{2})\cdot \cdots \cdot m_{n}(A_{n})$$

here, K represents the conflict measure of the belief functions.

#### 3.6.2. The Multi-Classifier Fusion Algorithm

**Figure 5:**
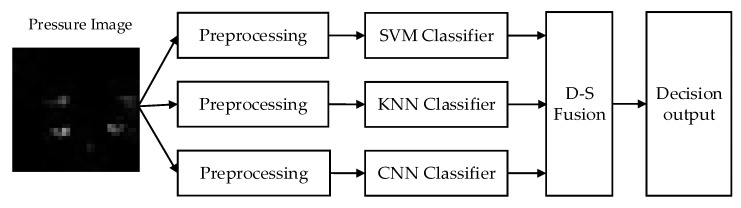
Framework of the multi-classifier (CNN classifier, support vector machine (SVM) classifier, *k*-nearest neighbor (KNN) classifier, and the Dempster–Shafer (D–S) evidence theory) fusion algorithm (CSK–DS).

To improve the recognition results, the proposed classification algorithm fuses multi-type classifiers based on D–S evidence theory. By virtue of their high classification effect in this paper, SVM, KNN, and CNN were selected for verification. Figure 5 shows the framework of the algorithm. First, the three classifiers were trained to obtain the classifier models. The outputs with high recognition rates from the three classifiers were then fused by information-fusion technology based on the D–S evidence theory. Finally, the standing posture was selected from among the fused target information by Dempster’s combination rule. 

## 4. Results

### 4.1. Dataset

**Figure 6:**
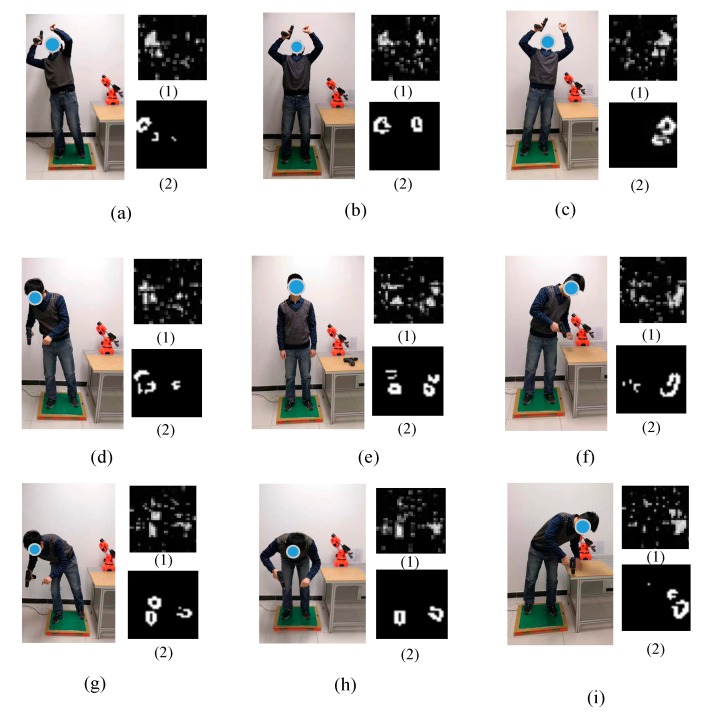
Standing postures evaluated in the experiment: (**a**) RBS; (**b**) BIS; (**c**) LBS; (**d**) RLS; (**e**) URS; (**f**) LLS; (**g**) RFS; (**h**) FLS; and (**i**) LFS. In each case, (**1**) is the original image and (**2**) is the image after Gaussian filtering.

Figure 6 displays a part of the raw dataset. Nine standing postures were collected from the 10 participants, so the dataset was divided into nine categories. Each posture corresponded to two images: the original image (1) and the filtered image (2). In the original image, only the grayscale image formed by the floor force can be seen, along with some clutter interference. After passing through a Gaussian filter, the interference pixels were removed from the original image and the sole outlines the visible. We collected two datasets: a threshold-filtered dataset and a Gaussian-filtered dataset. Eight times in total, we randomly selected 80% of the dataset for training, and retained 20% as the test set.

### 4.2. Experimental Results of CNN

**Figure 7:**
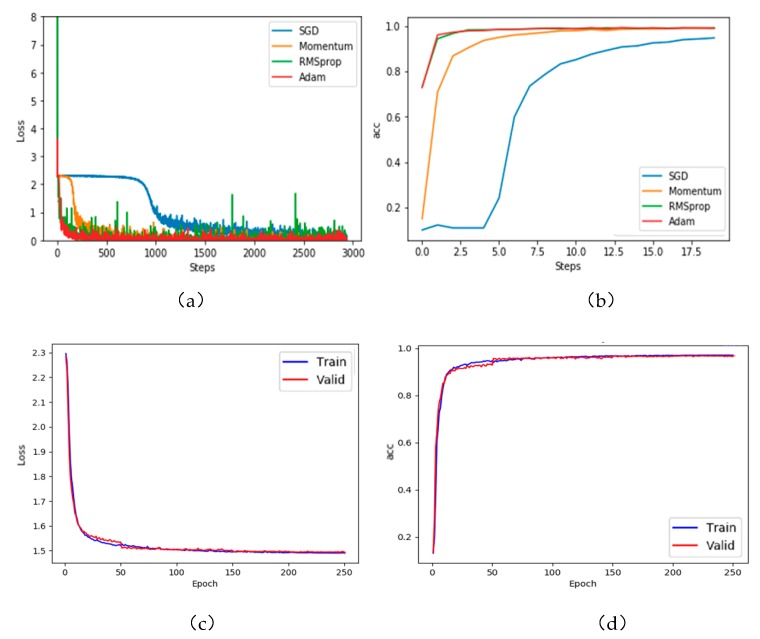
(**a**) Loss function curves of the training set. (**b**) Recognition rates. (**c**) Loss functions and (**d**) accuracies of the training and test set obtained in the fivefold cross-validation.

The structure of the neural network used in this experiment is shown in Figure 4. As mentioned in the previous subsection, the dataset was randomly divided into training and test sets at a ratio of 0.8:0.2. The learning rate was set to 0.0001. After each training, the training and test sets were reselected at random until eight training-test dataset pairs had been formed. Our human standing- posture-recognition model was trained with four optimization algorithms (stochastic gradient descent (SGD), Momentum, RMSprop, and Adaptive Moment Estimation (Adam)). Panels (a) and (b) of Figure 7 present the loss functions of the training set and the recognition rates, respectively, obtained by the optimization algorithms. The evaluations were performed by fivefold cross-validation. The loss functions and accuracies in the cross-validation test are displayed in panels (c) and (d) of Figure 7, respectively.

As evidenced in Figure 6a,b, the Adam algorithm was more effective than the other optimization methods. Referring to the literature [43], the recognition rate of Adam tends to stabilize after approximately 10 epochs. The recognition rate on the test set was 96.16%. 

To optimize the classification results, we adopted a new optimization method that combines Adam optimization with SGD optimization. First, the Adam optimization method adaptively adjusts the learning rate until the network converges quickly. Next, the trained model with the minimal learning rate is fine-tuned by SGD. The SGD method re-optimizes convolution layer C4 and the fully connected layers (F6, F7, and F8) in Figure 4, but maintains the parameters from the input layer to pool layer S3. The initial recognition rate was set to 0.0001. The recognition rate of the final model on the test set was 96.412%.

The effects of data augmentation, BN, and step-by-step training on the network recognition rate and training time were experimentally evaluated without data augmentation, with an optimization method, and without BN, respectively. The experimental results are shown in Table 2.

### 4.3. Comparison with Other Classifiers

The performance of the proposed method was evaluated in comparison studies of several common classifiers using six common classification methods: SVM, KNN, RF, DT, NB, and BP. All classifiers were trained on the nine standing postures in the training dataset, and their accuracies were determined on the same test data. Table 3 lists the classification rate of each classifier on the test data with threshold filtering. The average classification accuracy ranged from 83.28% (in the BP network) to 99.96% (in CNN). The classification rate of CNN ranged from 96.04% to 96.86. The accuracy of SVM using the RBF kernel ranged from 92.55% to 97.72%. The DT and NB classifiers were closest in accuracy to the SVM and BP classifiers, respectively. Meanwhile, the accuracies of the KNN and RF classifiers were only slightly different. Using CNN, SVM, and KNN, we finally constructed the basic probability-assignment function for the initial recognition results of the target and fused these results with Dempster’s combination rule at the decision level. The fusion-recognition method proved effective and robust, achieving a recognition rate of 99.96%.

Table 4 shows the classification rate of each classifier on the test data with Gaussian filtering. The recognition rates were lower than in Table 3, with average classification accuracies ranging from 75.08% (in NB) to 90.47% (in CNN). Furthermore, Table 3 and Table 4 show that the classification rate was highest in the CNN classifier and lowest in the NB classifier.

**Figure 8:**
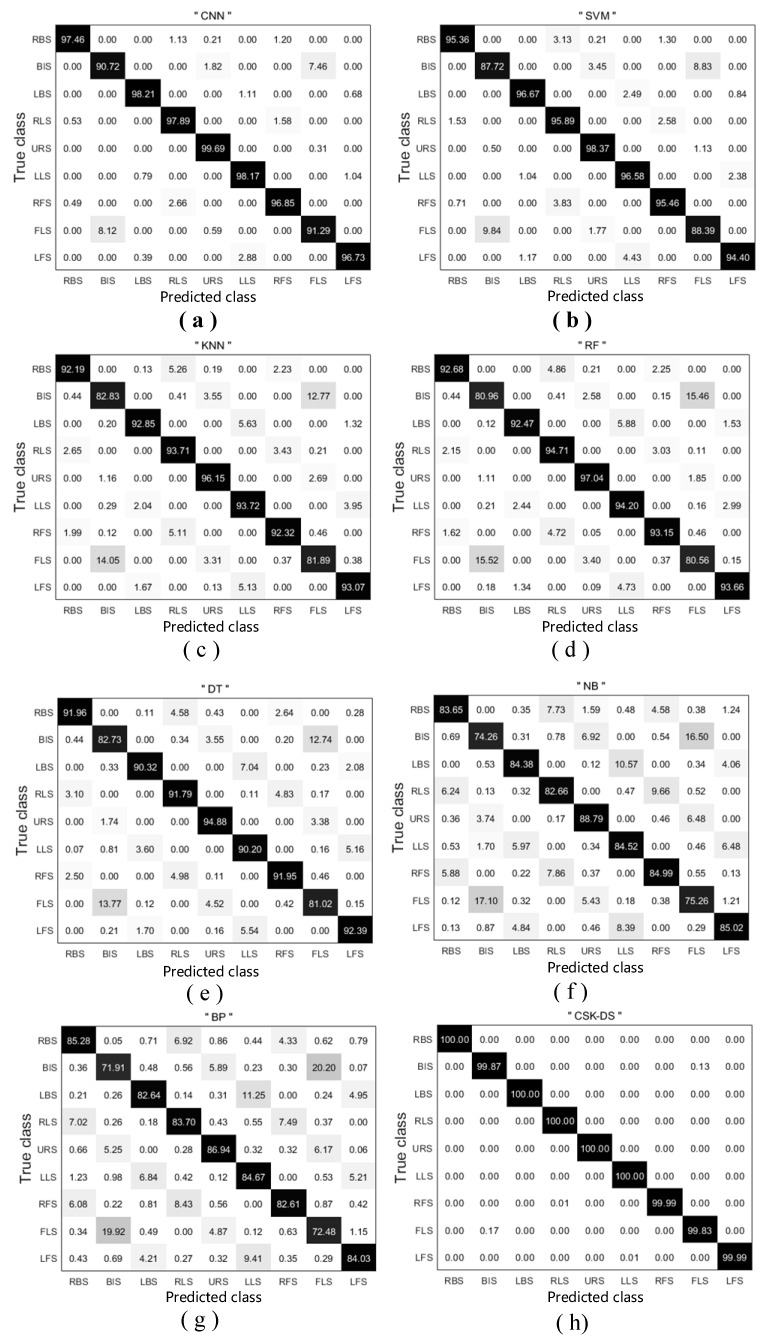
Confusion matrix of each classifier on subject F: (**a**) CNN; (**b**) support vector machine; (**c**) *k*-nearest neighbor classifier; (**d**) random forest classifier; (**e**) decision tree classifier; (**f**) naive Bayes classifier; (**g**) backpropagation neural network; (**h**) D–S fusion method.

Figure 8 shows the confusion matrix of each classification rate in the case of participant F, which was well-classified by all classifiers. Whereas most classes categorized by the CNN were labeled as “true”, the classification rates were obviously lower for BIS and FLS than for the other standing postures. BIS was frequently classified as FLS and vice versa. The same situation appeared in multiple classifiers. Furthermore, RBS was occasionally misclassified as RLS or RFS, and LBS was occasionally misclassified as LLS or LFS. The classification rate of URS was the highest in all classifiers. The theory of evidence fusion improved the recognition rates, especially those of BIS and FLS. The average recognition rate over all attitudes exceeded 99.8%.

## 5. Discussion

The objective of this paper was to classify the standing postures in an HRC system using the CNN algorithm and a data fusion method. Comparing the SPCS results with actual actions confirmed the significant benefits of the SPCS application. In particular, the SPCS recognized the standing positions of human workers in standing-only situations and provided the precise coordinates of the human body relative to the operating platform. Moreover, the standing-posture recognition of SPCS was valid at 40 Hz. The SPCS avoids the instability caused by occlusion of the camera sensors in camera-based pose recognition. It also avoids privacy violations of images, which sometimes draw complaints. The following discussion covers the two components of the proposed system.

### 5.1. SPCS

Our proposed system classifies standing postures through a thin-film pressure-sensor matrix. In contrast, the existing SPCS studies are based on textile or other force-sensor matrices. Our experiments confirmed that the SPCS can detect very small pressure differences, for example, gentle changes of body postures. The pressure detected by a single sensor in the floor depends not only on the overall weight and contact area, but also on the shape, softness, and roughness of the contact surface. The pressure matrix covers a large range of measurements expected in typical HRC systems, and is sufficiently sensitive to detect the active states of most human standing postures.

The signal generated by the pressure floor is expressed by the pressure matrix, which gives the surface-pressure distribution at any given time. Analogously to a charge-coupled device camera, we obtain a data stream of pressure images. The data form of the pressure-perception matrix is similar to that of images, but the signal processing of pressure images differs from that of “normal” images as described below. 

(1) The pressure-matrix data obtained from the floor reflect the changes in resistance values of the thin-film sensors. Under relative pressure, a single-point sensor in the pressure floor registers a change of resistance, which is related to a pixel in the pressure image. Due to the structure of the pressure-film sensor, the impedance between adjacent sensors can reach dozens of MΩ. Therefore, when a sensor is pressed, the nearby sensors are unaffected, so any change in a single-pixel value will not change the adjacent pixel values. This characteristic has been improved by referring to the literature [25]. 

(2) The image information produced by the pressure floor maps the data of the pressure-sensor matrix to the gray-image information, which differs from the usual computer-vision application. During data acquisition, the key data information usually changes at a fast rate (here, the refresh rate was 40 Hz) to capture the real-time movements of a human body when standing. Because the acquisition controller uses a 12-bit ADC, the pressure-image data rapidly responds to slight pressure changes. 

### 5.2. Standing-Posture Classification Method

Using a CNN classifier, our proposed method recognizes standing postures from the pressure distributions captured by the pressure-sensing floor. As shown in Table 5, most studies on posture classification used many more sensors than our proposed system. For example, Cheng et al. embedded multiple sensors in a textile-sensor mat to classify various sitting postures [25], but their classification accuracy was not significantly higher than ours. The method of Costilla-Reyes et al. uses a CNN for learning spatial footstep features and a nonlinear SVM model for footstep recognition [34]. Zhou et al. presented a person-identification approach based on the morphing of footsteps measured by a fabric-based pressure-mapping sensor system [35]. The proposed method classified nine typical postures of subjects standing on the perception floor.

The standing postures on the sensing floor were distinguished by seven classifiers (CNN, SVM, KNN, RF, DT, NB, and BP neural network). Among these classifiers, CNN most effectively classified standing postures from the patterns of a sensor matrix exclusively positioned on the floor plate, with an average classification rate of 96.41%. Furthermore, the CNN results were statistically different from the average results of the other classifiers. On the Gaussian-filtered dataset (Table 4), the average accuracies of the classification results were lower than when the dataset was filtered only by the threshold. Unlike image processing of the MNIST dataset (a database of handwritten digits), the classification of human-posture pressure images must obtain not only the image shape (as in handwriting recognition), but also the specific pressure distribution. After Gaussian filtering, the recognition rate was degraded by loss of important information which was retained in the threshold-filtered dataset. As revealed in the confusion matrices, certain standing postures (such as URS) were accurately detected whereas others (such as BIS and FLS) were sometimes misclassified. The images of BIS and FLS were similar, especially after Gaussian filtering (c.f. panels (b) and (h) in Figure 6). To exploit the unique advantages of the above classifiers, we fused different methods based on evidence theory. The experimental results showed that the D–S fusion algorithm further improved the classification accuracy. After fusion, the recognition rate of several postures reached 100%, and even postures with low recognition rate (i.e., with high feature similarity) were recognized with 99.8% accuracy. According to these results, the data fusion method fully utilizes the advantages of each classifier and further improves the certainty and robustness of the posture classification.

Although the above methods have achieved some results, however, the above methods still have their limitations. These include that all the data and tests are based on the static pressure data of the human body standing on the pressure floor. During the CNN experiment, we randomly assigned three data sets (training/test/validation sets) after data augmentation. In theory, the deformed image may appear in the test set and the final test result may be higher. The challenge of our method is that the human body is in a state of continuous activity in the actual interactive scene. Some exceptions, such as single foot support and the squat position have not been considered.

## 6. Conclusions

This paper applied deep learning to a human–robot collaboration (HRC) system. The standing-posture classification system (SPCS) recognizes typical poses in HRC by thin-film pressure sensing, a novel sensing modality. We developed the general hardware architecture of SPCS and a CNN classification method. We also demonstrated the feasibility of SPCS in seven representative classification methods and a fusion method. In an experimental case study, the SPCS achieved standing-posture recognition accuracy of >96% in the CNN classification, and 99.96% in the fusion method. Based on the recognition probability of the three classifiers (CNN, SVM, KNN), the CSK–DS algorithm achieved a higher recognition rate without reducing the output frequency (40 Hz). At the same time, the robustness of the SPCS was further improved. The human standing postures were detected while the subjects wore their shoes, which better reflects the real situation than posture prediction without shoes (as done in previous studies). Moreover, predicting human-body postures from the pressure distributions on the pressure floor does not invade the privacy of workers. This work will provide the basis of a high-performance HRC system. In this study, we confined our technique to static images of human standing. Future studies will dynamically test our system on humans working with a robot system in real-world settings. 

## Figures and Tables

**Table 1:** Body conditions of the participants in the experimental study.

Participant	Sex	Height (cm)	Weight (kg)	Shoe Size (EU)
A	male	168	60	43
B	male	172	71	39
C	male	181	80	42
D	male	179	96	44
E	male	178	75	41
F	male	171	55	40
G	male	166	50	39
H	male	178	65	43
I	female	165	45	37
J	female	162	41	36

**Table 2:** Recognition rates and training times of different methods.

Recognition Method	Recognition Rate (%)	Training Time (s)
Without data augmentation	92.548	235
Without BN	94.448	6849
Adam + BN	96.126	1233
SGD + BN	96.605	1154
Adam + SGD + BN	96.412	1161

**Table 3:** Classification rates of test data with the threshold filter.

Classifier	CNN	SVM	KNN	RF	DT	NB	BP	CSK–DS
1	**0.9610**	0.9524	0.9212	0.9285	0.9089	0.8458	0.856	**1.0000**
2	**0.9612**	0.9576	0.9054	0.9293	0.8908	0.8165	0.825	**0.9988**
3	**0.9604**	0.9255	0.9155	0.9192	0.8968	0.8228	0.8266	**1.0000**
4	**0.9636**	0.9351	0.9069	0.9086	0.8854	0.8462	0.824	**1.0000**
5	**0.9622**	0.9298	0.9124	0.9198	0.8862	0.8431	0.8166	**1.0000**
6	**0.9686**	0.9466	0.9026	0.9254	0.8788	0.8196	0.834	**1.0000**
7	**0.9642**	0.9522	0.9266	0.9171	0.9092	0.8347	0.8514	**0.9999**
8	**0.9618**	0.9772	0.9198	0.9254	0.8826	0.8412	0.8289	**0.9982**
Average	**0.9628**	0.9445	0.9138	0.9216	0.8923	0.8337	0.8328	**0.9996**

**Table 4:** Classification rates of test data with the Gaussian filter.

Classifier	CNN	SVM	KNN	RF	DT	NB	BP	CSK–DS
1	**0.9044**	0.8657	0.8246	0.8274	0.7903	0.7397	0.7443	**0.9975**
2	**0.9032**	0.8546	0.8341	0.8183	0.7842	0.7596	0.7564	**0.9975**
3	**0.9065**	0.8498	0.8167	0.8195	0.7605	0.7483	0.7345	**0.9978**
4	**0.9047**	0.8812	0.8054	0.7941	0.7862	0.7455	0.7697	**0.9979**
5	**0.9021**	0.8368	0.8132	0.8129	0.7917	0.7631	0.7487	**0.9978**
6	**0.9066**	0.8439	0.8055	0.8217	0.7791	0.7368	0.7661	**0.9981**
7	**0.9005**	0.8567	0.8371	0.8153	0.7849	0.7459	0.7546	**0.9983**
8	**0.9091**	0.8633	0.8174	0.8044	0.7924	0.7682	0.7468	**0.9983**
Average	**0.9047**	0.8565	0.8195	0.8142	0.7836	0.7508	0.7526	**0.9979**

**Table 5:** Comparison between the reported studies and proposed methods.

Author	The Sensor Type and Number	Sensor Position	Number of Subjects	Classification Method	Number of Postures	Refresh Rate	Accuracy
Cheng et al. [25]	textile pressure-sensing matrix 80 × 80	On floor	11	KNN	7	40 Hz	78.7%
Costilla-Reyes et al. [44]	piezoelectric sensors 88 × 88	On floor	127	CNN + SVM	3	1.6 kHz	90.60%
Zhou et al. [35]	fabric sensor mat □ 120 × 54	On floor	13	RNN (Recurrent Neural Network)	person identification	25 Hz	76.9%
Zhang et al. [45]	Force Sensing Resistors: 504 × 384	On floor	2	Mean-Shift Clustering	7	44 Hz	95.59%
Proposed method	Pressure Thin Film Sensor □ 32 × 32	On floor	10	Improved CNN	9	40 Hz	96.41%
D–S fusion	CNN–SVM–KNN		10	D-S theory	9	40 Hz	99.96%
